# Essential Elements of Success in Dental Practice

**Published:** 1876-09

**Authors:** K. B. Davis


### ESSENTIAL ELEMENTS OF SUCCESS IN DENTAL PRACTICE.

BY K. B. DAVIS, D. D. S.

*Read before the Iowa State Society.*

Doubtless the fondest hope, the most cherished desire, the most earnest day dream of every professional man, is that he may in every respect attain or achieve the very highest success. This desire, perhaps, animates alike the breast of every member of all professions, whether he belongs to the legal, theological, medical or dental professions. Yea, I might say that this desire is universally entertained by men in every vocation in life. The business man, the speculator, the capitalist, the agriculturalist and the mechanic are all eager to achieve at least pecuniary success.

Seeing then, that success is so eagerly desired by men in all the pursuits of life, we need not wonder at its manifestations in the dental profession.

There is nothing, it seems to me, more evident than that every thing in the universe is governed by established laws. And as in the planetary system myriads of orbs revolve in resplendent order around one common center, directed in all their course by fixed and unalterable laws, so complicated that the slightest variation on the part of any one body must end in universal, "wreck of matter and crash of worlds;" so in human life, every cause produces its legitimate effect; every action is followed by its legitimate consequence. Thus am I led to believe that our success is, to a very great extent, dependent upon the possesion of certain elements, and that our failures are caused from a want of these elements.

1. Education.—First in the list of essential elements of success is education. This is of fundamental importance to any one that aims at any thing like success, and underlies all scientific practice. Any one, before entering the dental profession, should be well assured that he has the natural, inherent fitness and the adaptation in order to the acquisition of skill as a manipulator. Both a good student and a good manipulator is important to constitute a successful dentist. Many in our profession are studious but, very poor manipulators; dependent sometimes upon improper training, but more frequently upon deficient natural endowment. By assiduous industry, patient perseverance and special advantages, this may, in some instances, be to a very great extent overcome, but in very many others this deficiency is utterly beyond remedy. There is another class who are good manipulators but are not good students; willing to perform any of the manual duties incident to a dental practice, but very little inclined to purely mental labor or study.

Both these classes are found in our profession, and in very considerable numbers too; but they are not as successful as they should be, nor, as they could be, if they would combine the very best trained manipulative ability, with the most thorough culture. In other words, to attain the highest results the operator should be able to perform with the hands, any and all operations according to the highest mental conceptions of what a perfect operation should be.

In order to achieve successful results what then should be in our aim in an educational aspect? What is education? It is not merely accumalating facts, principles, or knowledge. These are important it is true; but they are only the instruments that are to be utilized in the future; but it also means to develop, draw out, strengthen and train the mental faculties so that the materials that may be treasured up may be employed to secure the most efficient success.

As in almost all other departments in human occupation superficiality has been, and is still, the bane of the dental profession. The wild folly is widely entertained that any body can practice dentistry. But this absurd idea must be eradicated from the minds of the American people. There must be more professional education, more intellectual discipline, more culture, more breadth and comprehensiveness in the masses of our profession, if they ever attain the successful results so much to be desired. The high and grave responsibilities of the profession must have recognition and acknowledgment. A position in our profession must be attainable only through gates as straight and ways as narrow as those that lead to the legal or the medical profession, for the science and practice of dentistry demand talent, capabilities, knowledge and culture, as great as any of these.

To make education as proficient an element of success as its capabilities will admit, it should be comprehensive, thorough and progressive. Broad enough to embrace not only dental science, but every department of medical science. This education, this self-training, this earnest mental application and devoted study for our general advancement and professional success should not be kept up for a year or so after commencing practice and then abandoned, but should be a continual earnest life business with us if we wish to succeed. Especially should operative dentistry, oral surgery, physiology, histology, pathology, chemistry and dental therapeutics be thorougly studied up to their present development. I do not mean by this that we should cumber the memory with difficult technicalities, but that we should become so familiar with them, that mere names would dwindle into insignificance. This knowledge should become such a part of our professional personality, that it ever can be held in reserve and thus can we call it up under any emergency.

Superadded to education as an auxiliary element of success, is literary culture. We are, as a profession, brought into close and intimate relations with the most refined, educated and cultured classes of society. This being true, every one who desires to secure this class of society as his patrons must possess literary culture. Every dentist needs it; it will make him a better as well as a more successful dentist. It gives him influence and greatly extends the sphere of his usefulness. A dentist should not be content to be a mere dentist. He should know something at least about literature and be able to converse well, act well, and appear well, not only in the dental office, or dental society, but also in the drawing room or the public assembly.

A man will be a better lawyer, a better physician, a better preacher, or a better dentist for having literary culture. The truth of this is so obvious as to need no argument. Indeed literary culture is needed by every professional man, because it is calculated to improve the taste, to refine the manners, to enrich the mind, to elevate the morals, to give more power, in short it will make one happier, wiser, better and more successful.

2. Skill.—This is pre-eminently an element of success to every operator who possesses it. Indeed, without skill there can be no success, but on the other hand continual failures and disappointments. "Skill, both in operating and remedial practice is principally acquired, and is the result of professional knowledge, extensive observation and practical experience. Then there are some who can never acquire skill, no matter how diligently and conscientiously they apply them selves; they seem to have no natural faculty for the acquisition of this much needed element of success."

Seeing then that they can never rise above the point of mediocrity as operators, the duty of such men is very plain. They should, as a matter of justice to their patients, themselves and the profession, hasten to transfer their labors to some other vocation or sphere of usefulness. "Then there are others who seem to have, in a very marked degree, this natural ability or aptitude for the acquisition of skill, and very soon make not only very fine operators, but are very successful in the treatment of oral diseases. Again there are others who seem at first to be deficient in natural ability or aptitude, that are able by a careful training of the hands and a conscientious and energetic mental effort to finally overcome this, and in time, to arrive at a very high degree of success in every department of practice." There is, perhaps, still another class who have the natural ability, if properly developed, to make very skillful operators, but who, from deficient education, and a want of proper manipulative training and accomplished models as guides in operating, their efforts are more frequently failures than successful. This class in our profession should immediately avail themselves of all opportunities for the proper cultivation of their minds, and of the requisite training of the hands to secure skill and success in manipulating. If this is done with a thorough will, and a conscientious and honest determination to succeed, the avenues of success will be found open; skill will be readily acquired, that ultimately brings with it rapid improvement and with all triumphant results and success.

In order to achieve the highest degree of success, we must not only be skillful manipulators, but we must also be skillful in the treatment of all pathological conditions of the oral cavity. Many seem to entertain the idea that skill in manipulating is alone necessary; and, consequently, they make little or no effort to acquire it in all departments of our profession. But if one desires true success in the highest degree he must be skillful, not only in operating, but in every department of practice.

3. Thoroughness.—The importance of this as an element of success in conservative practice can not be too highly insisted upon. "Thoroughness is an application of our highest skill; and though it may be considered as having been embraced in the consideration of the subject of skill, as necessarily a part of it, yet its importance demands especial notice, We may have every other requisite for success, yet if this is lacking we can not hope to attain the highest results, unless this element is ever prominent in every operation that we perform. The thorough preparation of cavities in filling teeth; the careful removal of all imperfect dentine and enamel from the margin of cavities; the smoothing and polishing of the edges; the careful packing of every piece of gold; the thorough finish given to the filling on every surface—in fact, thoroughness in every step of the operation from its commencement to its completion, is essential to the highest success."

In the removal of pulps; the treatment of alveolar abscess; removing deposits of tartar; regulating the teeth; treating any of the abnormal conditions of the oral cavity—in fact, every thing we are called upon to do, should be done in a conscientiously thorough manner, if we desire to secure the highest and most successful results. When we have made this the rule of our life in practice, then we shall receive daily testimony that our operations are conserving the dental organs, of duty faithfully performed, which shall reward us for our efforts and stimulate us to pursue with greater and growing earnestness the only true path to success.

4. Patience and Perseverance.—Any one of extended experience in our profession should readily acknowledge the importance of these elements in securing successful results. They are very requisite to success in any vocation; but they are pre-eminently so in the dental profession. We need patience and perseverance to enable us to succeed with our nervous patients. This is especially demanded in our operations upon the teeth of children. We need it in excavating and preparing difficult cavities; in adjusting the rubber dam in extremely difficult places; in starting and inserting many difficult or complicated fillings; in treating alveolar abscesses; in correcting irregularities of the teeth; indeed, I may say, that a daily and continual exercise of these elements under almost innumerable circumstances is demanded by the operator who aims to make his efforts as thorough and perfect as possible. A want of patience and perseverance has often caused failures when other circumstances were all favorable to success. There is an old Latin proverb which says, "hasten slowly," It is rarely that we find two words which express so much, or contain more food for thought. It would, perhaps, be well for every one of us to make this our motto in our daily practice, in order to secure the best results.

In every instance where these two elements are wanting, the operator should at once set about a proper course of cultivation to secure them, in a sufficient degree, to meet any emergency that may ever arise in an extended practice.

5. Industry.—This is another one of the essential elements of success in any vocation in life; but none the less so in our profession. All other things being equal, the industrious operator will always be more successful in his results than those who are destitute of this element. The industrious operator is not continually seeking the easiest methods of doing every thing, irrespective of successful results; whilst those who are wanting in this essential, are always disposed to get along with the least possible physical or mental effort, let the results be to the patient what they may. One may possess all the above mentioned requisites for success, and if yet lacking industry, he will fail for the want of some thing to bring these into action. There is, perhaps, a very large class in our profession who are groping along in the old ruts and grooves of many years ago; constantly meeting with failures and disappointments, that could, if they possessed the requisite energy and industry, become very successful and creditable operators.

We owe much at the present time to the industry and persevering of many noble and magnanimous spirits in our profession for its present efficiency and exalted position; but there is yet much to be done. There are many scientific problems that need investigation and solution; some erroneous theories and methods of practice to be corrected; much laborious effort is needed in the education and elevation of the entire profession to a higher plane of usefulness, and the accomplishment of greater and more permanent results. To accomplish all of this, we need an untiring industry and energy that will never permit us to cease our efforts until these desirable results are fully realized.

There are many noble examples on record of what may be accomplished by patient industry and energy. It was industry that made Elihu Burritt one of the finest linguists of this or any other age; it was this that made Dr. Franklin a philosopher and a statesman; it was this that enables Dr. S. D. Gross to accomplish so much literary labor amidst a large and lucrative surgical practice; it was this that enabled Disraeli to work out such an eminent public career. His first achievements were in literature; and he reached success only through repeated failures. As an orator, too, his first appearance was a failure. Though composed in a grand and ambitious strain, every sentence was hailed with "loud laughter." But he concluded with a sentence that embodied a prophesy. Mortified at his failure he exclaimed: "I have begun several times many things and have succeeded in them at last. I shall sit down now, but the time will come when you will hear me." The time did come, and the manner in which he succeeded in commanding the rapt attention of the British House of Commons, affords a striking illustration of what industry, determination and energy will do, for Disraeli earned his position by dint of patient industry. He worked patiently for success, and it came, but slowly; then the House laughed with him instead of at him.

Let these examples stimulate us to renewed efforts in our professional career, and may we, by dint of perseverance and industry work out success for ourselves and for the profession of our choice.

6. Professional Integrity.—Perhaps no one will deny that every dentist should be a man of virtuous principles, pure life and stainless reputation. No one of ignoble character, depraved principle, or corrupt practices, can ever expect success in our profession. Aside from the irrevocable obligations written and unwritten, resting upon all men to be good and true—obligations which all feel, and from which none can escape; and aside from all the considerations of the exquisite and exalted and personal happiness that springs from conscious rectitude of heart and life there is in the case of dentists, a positive and enormous element of success arising from the consciousness of genuine nobility of soul and integrity of life. Deny it who may, it is nevertheless true, that other requisites for success being substantially equal, the operator of genuine integrity will always secure better and more permanent results than the operator that is destitute of this qualification. The one possessing integrity is actuated by a higher, purer and nobler motive than simply the acquisition of his fee. It is his earnest desire and determination to bestow his highest skill on every case without regard to his remuneration in mere dollars and cents. On the other hand the dentist who is wanting in integrity is actuated by no higher motive than to secure his fee in the easiest or by the shortest way known to him, irrespective of the results of his operations in a conservative point of view. The honest operator seeks always under all circumstances the highest good of his patients by the use of the best materials, by adopting the most approved methods of operating; by giving to his patients such instructions as will tend to preserve the dental organs in the future. Integrity is requisite even if one has no higher motive than mere pecuniary success. The old maxim, "Honesty is the best policy," is as true in regard to the practice of our profession as in any other pursuit in life. Seeming to be honest is not enough; this goes far and does much, but there is that in the genuine which can not be counterfeited to the deception of others, even while to the mere seemer of integrity there is ever present to his every introspective glance, failures, desolate wastes, and to his patients bitter regrets and disappointments. Can it be that in the perpetual shadow of such a back ground, the starless night of such a want of integrity, the dentist's soul will not be tinged, his work affected, his powers lessened.

7. A spirit of progressiveness.—I think that this may be safely classed as an element of success, when we take into consideration the fact that it is a leading characteristic of all the most eminently successful operators in our profession. An operator that has this progressive spirit is ever on the alert for any improved methods of operating that give undoubted evidence of greater success; for any superior mode of treating the diseased conditions of the oral cavity that are calculated to secure quicker and better results than by his old plans of treatment. The progressive operator is never satisfied with his present attainments; but is ever ready to avail himself of any means that will render his operations more nearly perfect. With this view ever prominent in his mind he procures and diligently studies all of the text books; takes and reads the dental journals; is a member of one or more dental societies, and works in every practical way for his own advancement and elevation and of the profession at large. The progress of dental science was never so rapid as at the present time. Investigation was never before so vigorously prosecuted—assisted as now by the appliances of research. Observation was never so acute; experimentation so thorough; reflection so profound or record so complete. New facts are in process of development, new theories being presented, and new modes of treating diseased conditions of the oral cavity suggested; new instruments and appliances are invented that are calculated to facilitate and better our operations and heighten our labors. No matter what may be the attainments of an operator to-day or what his methods of practice if he does not keep pace with this rapid progress of the profession he will in a short time be far behind it, both in theory and in his method of operating. He may meet his own expectations in regard to his results, for they will necessarily not be very great; but he will fail to meet the increasing demand of an enlightened public, and the demands he justly ows to his profession. He will soon lose all professional feeling and interest in his profession. "Every man," said Webster, "owes a debt to his profession." By this, we understand that every man is under obligation to do what he can for the elevation of his profession. We would then advise all dentists who wish to make success sure to unite heartily in every effort and plan designed for the good of our profession. By thus doing, our own personal profit and advancement will be secured.

Who of us is satisfied? Nay, who of us comparing the actual with the possible—the present with the hoped for, and the should be and the may be in our profession, is not ready to labor on until the bright anticipations of the future are fully realized.

Let us then work on, feeling a just pride in our calling, and with a determination that will succeed in spreading the truths of dental science over the land, and whispering in their ears these stirring and beautiful lines of Longfellow:

"Lives of great men all remind us
We can make our lives sublime,
And departing leave behind us
Footprints on the sands of time.

Let us then be up and doing
With a heart for any fate;
Still achieving still pursuing,
Learn to labor and to wait."

